# Low-dose patterning of platinum nanoclusters on carbon nanotubes by focused-electron-beam-induced deposition as studied by TEM

**DOI:** 10.3762/bjnano.4.9

**Published:** 2013-02-04

**Authors:** Xiaoxing Ke, Carla Bittencourt, Sara Bals, Gustaaf Van Tendeloo

**Affiliations:** 1 EMAT, University of Antwerp, Groenenborgerlaan 171, 2020 Antwerp, Belgium; 2 Chemistry of Interaction Plasma Surface (ChiPS), University of Mons, Place du Parc 20, 7000 Mons, Belgium

**Keywords:** carbon nanotubes, FEBID, nanocluster, platinum, patterning, radiation-induced nanostructures, TEM

## Abstract

Focused-electron-beam-induced deposition (FEBID) is used as a direct-write approach to decorate ultrasmall Pt nanoclusters on carbon nanotubes at selected sites in a straightforward maskless manner. The as-deposited nanostructures are studied by transmission electron microscopy (TEM) in 2D and 3D, demonstrating that the Pt nanoclusters are well-dispersed, covering the selected areas of the CNT surface completely. The ability of FEBID to graft nanoclusters on multiple sides, through an electron-transparent target within one step, is unique as a physical deposition method. Using high-resolution TEM we have shown that the CNT structure can be well preserved thanks to the low dose used in FEBID. By tuning the electron-beam parameters, the density and distribution of the nanoclusters can be controlled. The purity of as-deposited nanoclusters can be improved by low-energy electron irradiation at room temperature.

## Introduction

Hybrid nanostructures consisting of carbon nanotubes (CNTs) decorated with metal nanoclusters enable access to various electrical and catalytic properties. Therefore, they are considered as building blocks for nanoscopic electronic devices [1]. In such hybrid nanostructures, metals are often deposited onto the CNTs by thermal evaporation [2–6] or wet chemistry [7], which results in a non-site-specific covering. However, when using such structures for nanodevice fabrication, specific sites of the CNTs need to be functionalized in order to create components with specific properties. For instance, in order to fabricate CNT contacts on electrodes, Pd is thermally evaporated onto both ends by using shadowing masks [8]. In earlier reports, it has been shown that Au nanoclusters can be site-selectively decorated on the CNTs by using a focused ion beam (FIB) and subsequent chemical treatment [9]. However, these approaches either involve several steps or require masks to perform the site-specific deposition. Therefore, a more straightforward strategy to perform site-specific metal deposition is desired. In this paper, we explore the use of focused-electron-beam-induced deposition (FEBID) to pattern CNTs with well-dispersed ultrasmall nanoclusters.

FEBID is a direct-write process where a focused electron beam is used to locally decompose a precursor gas that contains a component such as a metal that is expected to be deposited on the substrate. The process can create nanostructures rapidly in a site-specific manner by scanning the electron beam precisely on the area of interest where the decomposition and deposition should take place. FEBID offers an efficient way of performing site-specific nanostructure deposition in a nondestructive way. A comprehensive review of FEBID can be found in the reviews of Randoph et al. [10], Huth et al. [11], Van Dorp et al. [12], Utke et al. [13–14] and Wnuk et al. [15], etc.

The applications of FEBID on CNTs are mostly focused on the formation of electrical contacts, and therefore much effort is being put into their electrical measurements and resistivity improvement, such as in references [16–17]. Nevertheless, the formation of different patterns of nanostructures at the CNT surface by FEBID is more challenging, since the direct-write process can produce various nanostructures beyond the simple formation of nanocontacts. For instance, by applying different precursor and deposition parameters, nanostructures of different dimensions, different chemical compositions and, thus, different properties can be formed at the CNT surface. Deposition of silicon [18], tungsten [19–20] and cobalt [21–22] nanostructures has been reported. A recent study has demonstrated the successful formation of binary Si–Pt nanostructures by FEBID [23].

Ultrasmall well-dispersed nanoclusters supported on CNTs are of most interest as the (electro-) catalytic activity can be increased [24–27]. The ability of FEBID to write ultrasmall nanostructures has been demonstrated by using scanning transmission electron microscopy (STEM) with an electron probe of 0.2 nm operated at 200 kV [28–29]. By using scanning electron microscopy (SEM)-assisted FEBID, Co nanowires of lateral size below 30 nm have been grown as well [30–32].

In this work, the site-specific deposition of Pt nanoclusters on CNTs by low-dose FEBID is presented. Electron tomography is performed to study the three-dimensional (3D) distribution of the as-deposited nanoclusters on the CNT surface. We observed the formation of a novel stripe-patterning of nanoclusters on the surface of the CNTs, which may open up new prospects of nanostructuring for applications in nanodevices dependent on the distribution of metal clusters. High-resolution transmission electron microscopy (HRTEM) and high-angle annular dark-field scanning transmission electron microscopy (HAADF-STEM) is used to study the morphology and distribution of the nanoclusters deposited by using different electron beam parameters. Although the as-deposited nanoclusters are composed of Pt and amorphous carbon, it is demonstrated that the amount of amorphous carbon due to the fragmentation of the organo-metal [(CH_3_)_3_Pt(CpCH_3_)], used as precursor for Pt deposition, can be reduced by using electron-beam irradiation with a low accelerating voltage as a post-deposition treatment.

## Results and Discussion

### 3D distribution of Pt nanoclusters around CNTs

**Figure 1:**
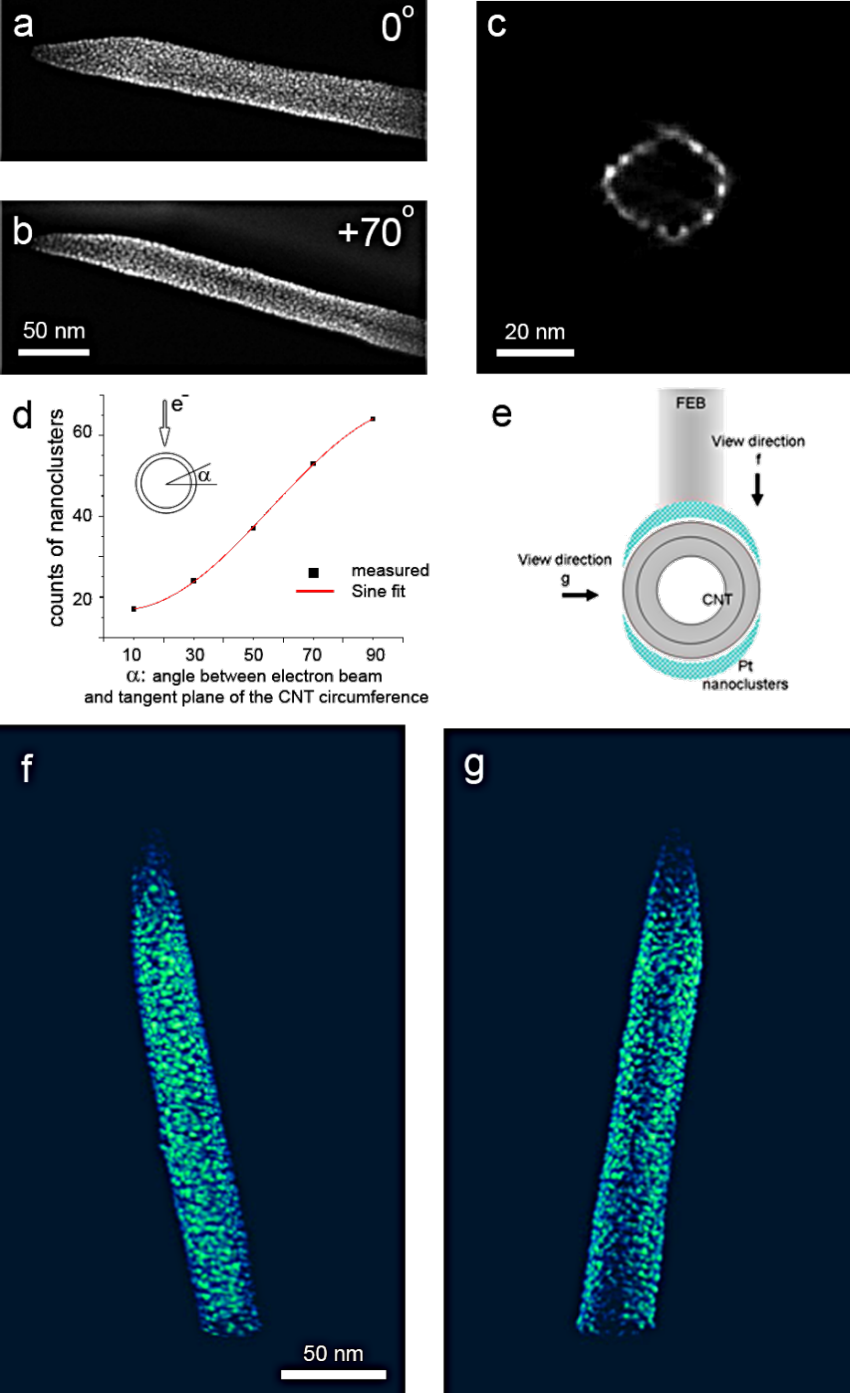
(a,b) HAADF-STEM images of the Pt deposited CNT at tilting angle of 0 and 70 degrees respectively. (c) A cross-section snapshot of the Pt deposited CNT from 3D reconstruction. Pt nanoclusters are shown as bright contrast. (d) Graph showing that the circumferential distribution of nanoclusters fits a sinusoid. (e) Illustration of the Pt distribution around the CNT. (f,g) Snapshots of the 3D reconstructed CNT along the viewing direction of f and g according to the illustration in (e).

When the CNTs are patterned by FEBID of Pt nanoclusters, the overall distribution of nanoclusters around the CNTs is one of the key factors to evaluate the effectiveness of the deposition process. As one of the most powerful and straightforward approaches to study the nanostructures in a 3D manner, electron tomography is performed to investigate the 3D distribution of as-deposited nanoclusters on CNTs. Figure 1 describes the 3D nanoclusters distribution study of a CNT decorated with Pt nanoclusters deposited by using an electron beam accelerated by 10 kV with a beam current of 0.54 nA; HAADF-STEM images from the tilt series at tilting angles of 0° and 70° and are shown (Figure 1a,b). In HAADF-STEM, the contrast scales with the atomic number *Z*, and therefore Pt nanoclusters yield a higher intensity in comparison to the CNT. Figure 1a,b demonstrates that the ultrasmall nanoparticles are well-dispersed across the whole surface of the CNT. Further 3D reconstruction of a tilt series of images (tilt range of ±70° with a 2° tilt interval, see Experimental section) confirms that the deposition has occurred all around the CNT resulting in a well-dispersed coverage. An orthoslice taken through the 3D reconstruction is presented in Figure 1c, illustrating the cross section of the as-deposited CNT. The dispersion of the Pt nanoclusters over the complete CNT surface verifies that the Pt nanoclusters are present on the entire surface of the CNT. A movie of the 3D reconstruction can be found in Supporting Information File 1.

We have observed that although the Pt nanoclusters are distributed uniformly along the long axis of the CNT, their distribution along the circumference is less homogeneous. A statistical analysis of the nanocluster distribution around the circumference of the CNT has been carried out based on the 3D reconstruction. As shown in Figure 1d, the distribution of nanoclusters has been quantified as a function of the angle α between the electron beam and the tangent plane of the CNT circumference. It can be seen from Figure 1d that the nanocluster distribution can fit into a sinusoid curve. The nonhomogeneous distribution of the nanoclusters can be demonstrated from the snapshots of the reconstruction shown in Figure 1f,g, where the density of nanoclusters at the CNT surface that faces towards the electron beam during deposition (Figure 1f) is higher than the one at the side of the CNT (Figure 1g). A straightforward and likely explanation can be given by considering the curvature of the CNT due to its tubular shape. It has been reported that besides the primary electrons, which are the direct incident electrons used in FEBID, the decomposition of the precursor molecules is to a large extent caused by the secondary electrons from the target surface as well [33]. The effect of the secondary electrons increases as the beam diameter decreases [29]. Due to the curvature at the CNT surface, both the density of primary electrons and the emission of secondary electrons reach a maximum at the surface perpendicular to the electron beam, and decrease for a surface with smaller angles to the primary beam. Therefore, the deposition has lower yields at the side surface parallel to the incident electron beam. As confirmed from the 3D reconstruction, the actual distribution of the as-deposited nanostructures can therefore be schematically illustrated in Figure 1e.

**Figure 2:**
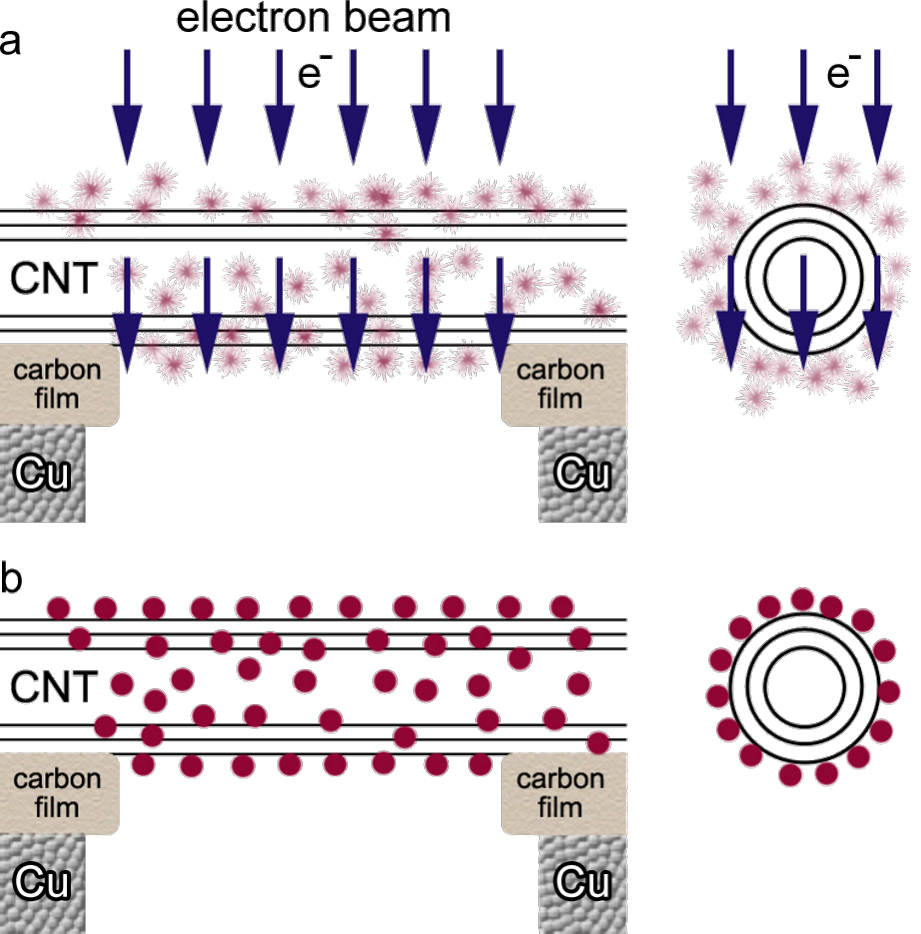
Illustrations of FEBID of nanoclusters on CNT. (a) Precursor gas is all around the CNT since the CNT is not attached to the substrate and is suspended above the support grid. CNT is viewed from both radial and axial directions. Electron beams transmit through the CNT as shown. (b) Pt nanoclusters are found all around the CNT as viewed from both radial and axial directions.

In addition, the 3D reconstruction shows that both the upper surface and the lower surface perpendicular to the beam have a similar distribution of Pt nanoclusters. This can be explained as follows: the CNT is placed on top of a holey carbon film supported by a Cu grid, i.e., the CNT is suspended rather than supported by a substrate. Therefore, when the precursor gas is released, the entire CNT surface is exposed to the precursor molecules (Figure 2a). When the electron beams scan the predefined area, the incident electrons and secondary electrons from the CNT decompose the precursor molecules in the vicinity of the CNT surface. Electrons not only decompose the molecules at the upper surface when they enter the CNT, but also decompose the molecules when they pass through the CNT and exit. Such electrons as well as the secondary electrons from the lower surface of the CNT decompose the precursor molecules in its vicinity as efficiently. Therefore, decomposition has its effect all around the CNT and results in Pt nanoclusters decorating both the upper surface and lower surface of the CNT (Figure 2b).

Demonstrating the ability of FEBID to deposit nanoclusters on both sides of the CNTs, we believe that the application of FEBID as a direct patterning approach can be extended to various electron-transparent structures, such as nanowires, thin films and graphene. Furthermore, taking into account that decomposition and further deposition is induced by interaction between electrons and materials, deposition can be tailored by tuning the accelerating voltage of the electron beam to control their transmission through a fixed thickness of target so as to realize the deposition on one or more facets.

### Stripe pattern of the Pt nanoclusters on CNTs

**Figure 3:**
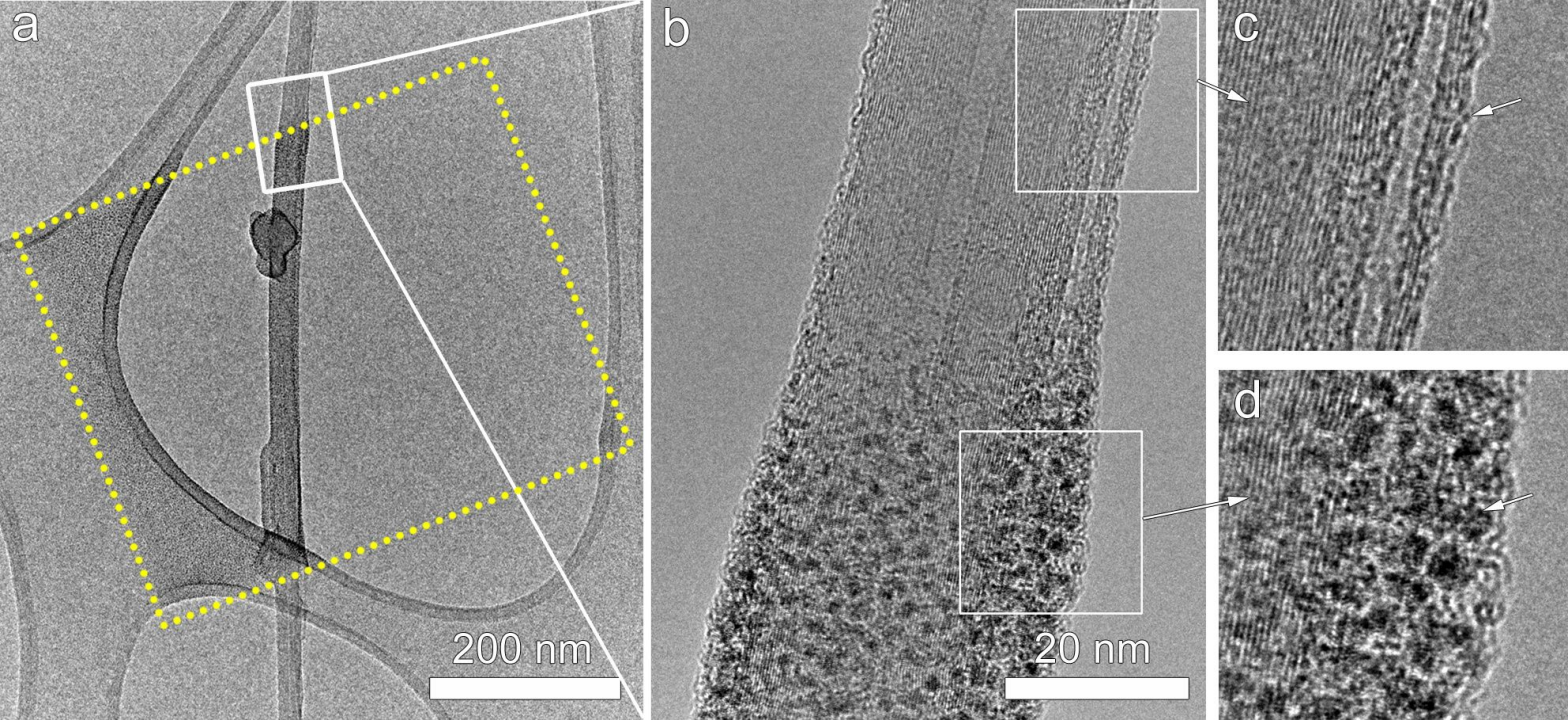
(a) TEM image of Pt nanoclusters deposited by FEBID on a CNT shows that site specific deposition has been achieved. The area indicated by the dotted lines is the predefine area during the deposition process. (b) Shows an enlargement of the rectangular dotted area in (a). The CNT structure has been preserved well during the deposition, as shown by HRTEM images (c–d).

When the deposition is performed in the first place, nanoclusters are expected to be confined in the preselected area only. A TEM image of the as-deposited nanostructure at a lower magnification confirms that the site-specificity has been accomplished (Figure 3). As shown in Figure 3a, the dashed box indicates the defined area during deposition and has a dimension of 400 nm × 500 nm. After the deposition, it can be seen that the deposited nanoclusters are strictly restrained in the predefined section of the CNT. The edge of deposited area is sharp and clean and leaves the rest of the CNT unaffected (Figure 3b). The unwanted proximity effect, which is noted to be related to a large dose and charging of the surface [12,34–35], is not seen at this scale. In fact, in this study a low dose is used and the CNTs have an “electron-transparent” thickness, thus the proximity effect can be ignored and the site-specificity is achieved at the nanoscale.

The irradiated and nonirradiated parts of the CNT are investigated by HRTEM (Figure 3c,d). We observe that the graphitic walls under the region covered by Pt nanoclusters (Figure 3c) show similar structure in comparison to the graphitic layers in the nonirradiated part of the CNT next to the deposited area (Figure 3d). This clearly reveals that the nanostructure of the CNT has been preserved during the FEBID process.

**Figure 4:**
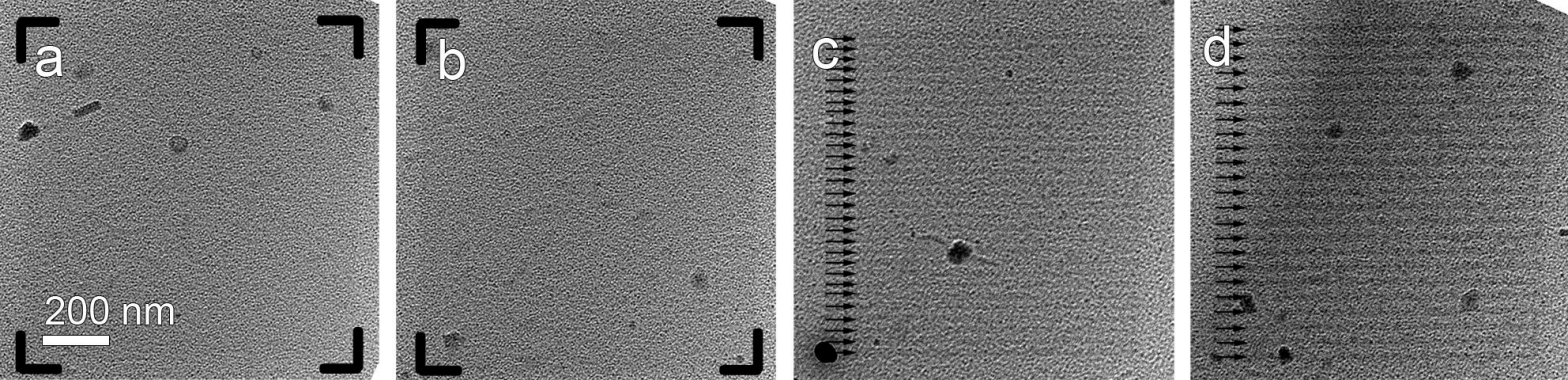
FEBID Pt on amorphous carbon at different defoci of the electron beam. (a) in focus, (b) at 4 μm defocus, (c) at 8 μm defocus and (d) at 10 μm defocus of the 30 kV 0.2 nA electron beam. Stripes with high densities are indicated by arrows and illustrations.

The deposition shown in Figure 1 and Figure 3 with uniform coverage of Pt nanoclusters on areas of interest is performed at the right focus of the deposition electron beam, i.e., the defocus value is 0. Nevertheless, if the deposition is carried out with the beam out of the focus, the as-deposited nanoclusters are no longer homogeneously distributed. This effect is demonstrated in Figure 4, where electron beams of different focus of 0 μm (i.e., in focus), 4 μm, 8 μm and 10 μm are applied for deposition onto an amorphous carbon film that was exposed to the Pt precursor gas. Detailed patterning parameters are described in Table S1 of Supporting Information File 2. It is obvious that the 0 μm and 4 μm defoci result in a uniform coverage of deposition (Figure 4a–b), whereas the 8 μm and 10 μm defocused electron beams lead to deposition of nanostructures in a stripe fashion (Figure 4c,d). In Figure 4c, the interstripe distance in the pattern is approximately 35 nm, which agrees with the pitch value of this patterning. In Figure 4d, the interstripe distance is approximately 45 nm, which also agrees to the pitch value during patterning (see Table S1, Supporting Information File 2).

The defocus of the electron beam is reported to have an influence on the volume growth rates [36]. Nevertheless, in this context where only low-dose deposition is studied, the growth rate may not be as important, whereas the formation of the stripe pattern can be attributed to regime shift during pattering along the X and Y axes. In the deposition using a 30 kV electron beam, the working regime is mainly electron-limited (please refer to the discussion of working regime and primary energy in the next section), which is reflected in the patterning along Y axis, where a high/low density of the Pt nanocluster distribution is presented. Nevertheless, along the X axis, the working regime is likely to be precursor-limited for the serpentine scanning strategy employed during this deposition.

**Figure 5:**
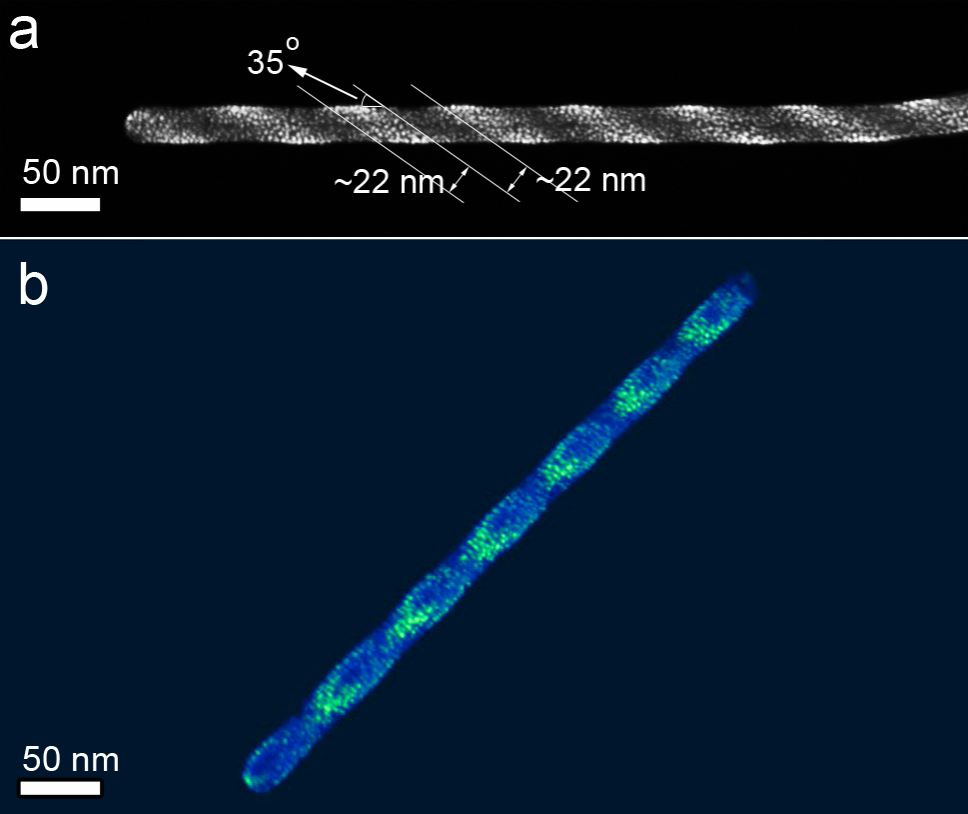
(a) HAADF-STEM image of a CNT with stripe-pattern of Pt nanoclusters from FEBID. (b) A snapshot from the 3D reconstruction of the nanostructure.

When we use a defocused electron beam for Pt deposition on CNTs, we observe that the Pt nanoclusters can be deposited in a switchable high/low density by fine-tuning the defocus of the electron beam. Figure 5a shows a CNT decorated with a high density of Pt nanoclusters discretely distributed at a regular spacing of approximately 44 nm. A HAADF-STEM tilt series of this nanostructure was performed and a 3D reconstruction was calculated. A movie can be found in Supporting Information File 3. A snapshot through the reconstructed nanostructure is shown in Figure 5b. The high-density stripes of the Pt nanoclusters are parallel to each other, and have an inclined angle of approximately 35° with respect to the long axis of CNT. This inclination can be tuned to any desired angle by positioning the CNTs relative to the scanning direction of the electron beam during FEBID. Furthermore, the size distribution of the as-deposited Pt nanoclusters is different from the uniform distribution seen in Figure 1. Larger nanoclusters are present in the striped area with higher deposition density, whereas smaller nanoclusters are present in the striped area with lower deposition density. The size distribution of the nanoclusters inside the stripes can be attributed to the regime shift as explained in the previous text. Furthermore, if we imagine a nanotube with a certain inclination angle to the electron beam, we can see that a varying defocus value during deposition can lead to a varying pitch in the stripe pattering along the long axis of the nanotube. The ability to pattern the nanostructure with switchable high/low density of nanoclusters provides new potential applications in tunable wetting, adhesion, catalysis and friction properties for nanodevices [37].

### Deposition parameters and the size distribution of Pt nanoclusters

Since the Pt deposition by focused electron beam is essentially a decomposition process of an organometallic precursor using electron beams, the morphology and dimensions of the as-deposited nanoclusters are largely related to the deposition parameter settings, including precursor and gas flow, the nature of the target being deposited, and the electron-beam parameters, etc. [34,38]. As one of the most important parameters, the influence of the different electron-beam settings on the deposited Pt nanoclusters is studied by changing the beam accelerating voltage (primary energy, PE) and dwell time, whereas the beam current is not varied in the current study.

**Figure 6:**
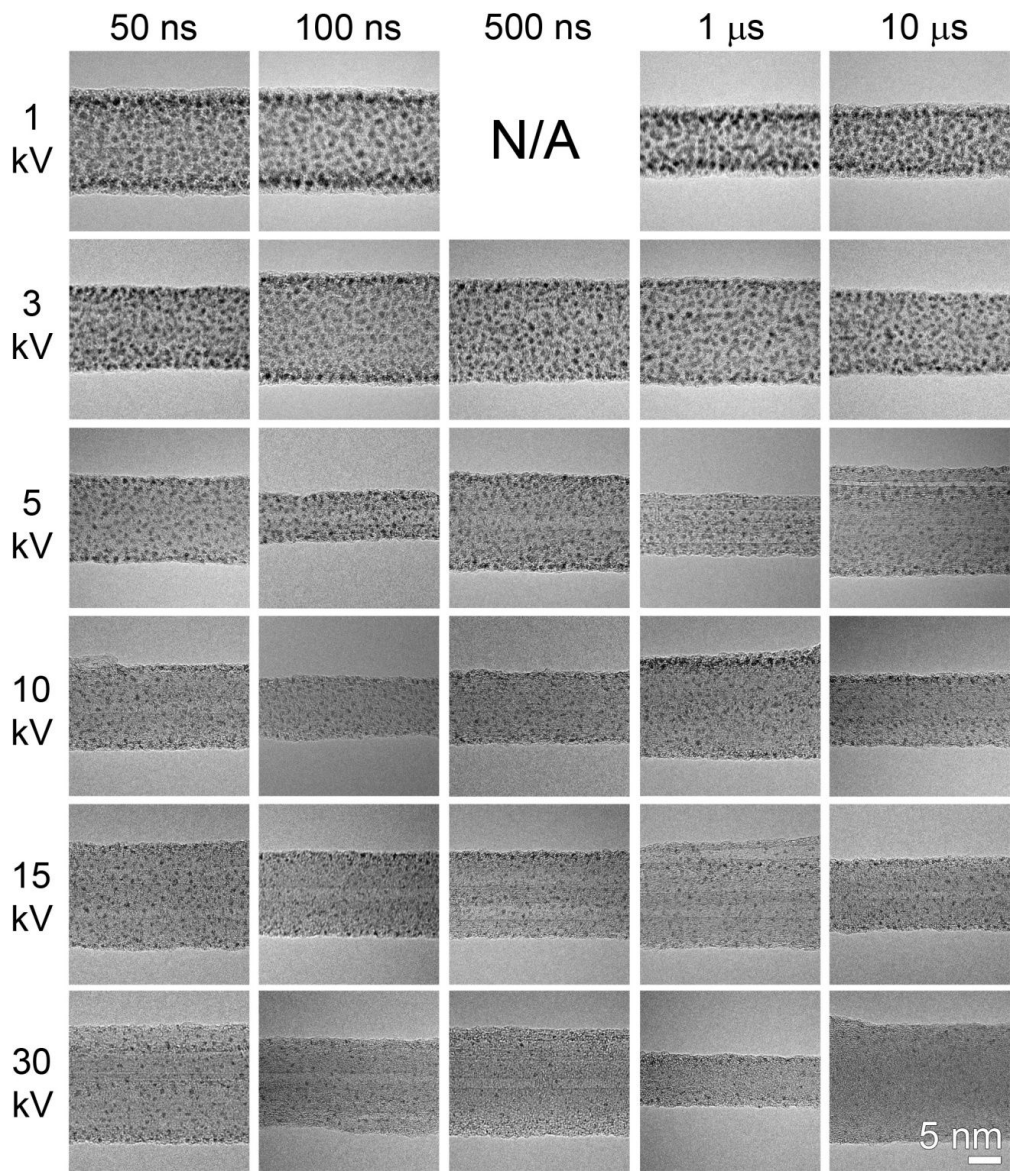
HRTEM images showing Pt nanoclusters deposited on CNTs by using different electron-beam settings. Beam energy is different as indicated in each row, whereas the beam dwell time is different as indicated in each column. The particular beam setting for the corresponding results are listed in Supporting Information File 2. For each beam setting, dwell times of 50 ns, 100 ns, 1 μs, 10 μs and 100 μs are applied for deposition.

Figure 6 summarizes the deposition of Pt for an increasing PE of 1 kV, 3 kV, 5 kV, 10 kV, 15 kV and 30 kV in each row. For each PE, different dwell times of 50 ns, 100 ns, 500 ns, 1 μs and 100 μs are applied for further comparison. Although the same electron dose is applied for each deposition, which means the same deposition time (2 s) as well as the same beam current is maintained (0.2 nA), the deposited nanoclusters demonstrate a different distribution. Comparing the nanoclusters in the same column in which PE is increasing and dwell time is not varied, it can be seen that their average size and lateral density decreases. The higher lateral density indicates a higher dissociation probability at lower PE, where PE contributes more to the deposition process. Simultaneously, lower PE results in a smaller interaction volume and therefore leads to increasing yields of secondary electrons and backscattered secondary electrons , which all contribute to the dissociation process.

When comparing the deposition in the same row in which PE is kept constant and dwell time is increased, it is noticed that the change in lateral density of the nanoclusters does not follow the same trend. When PE is 1 kV and 3 kV, the deposited nanoclusters have the same high density for all dwell times from 50 ns to 10 μs. Nevertheless, when PE is 15 kV and 30 kV, the nanoclusters show the same low density in the whole range of dwell times from 50 ns to 10 μs. Only when PE is 5 kV and 10 kV do the deposited nanoclusters demonstrate a decrease in the lateral density when the dwell time is increased from 50 ns to 10 μs.

It has been noted before that the precursor regime is largely dependent on the beam dwell time. Short dwell times lead to an electron-limited regime, whereas longer dwell times lead to a precursor-limited regime [10,39]. This well-known effect is reflected in the deposition results by a PE of 5 kV and 10 kV, where nanoclusters show a decrease in lateral density upon increasing dwell time. However, when a lower PE is used for the deposition, the rates of deposition are increased due to the higher number of potentially dissociating electron species (primary, secondary, backscattered, etc.), and therefore results in a working regime which is mainly precursor-limited, where the lateral density of deposited nanoclusters is higher. In the case of higher PE, a lower contribution of dissociating electron species results in a shift of working regime to electron-limited and thus shows lower nanocluster density.

### Improving the crystallinity of the carboneous matrix around Pt

In an ideal FEBID process, the electron beam is supposed to decompose the organoplatinum molecules completely, leaving only the metal atoms on the scanned area, and the volatile by-products should leave the surface. However, in an actual deposition process, the decomposition of the precursor gas is usually incomplete and residual fragments of the precursors as well as of residual gases in the deposition chamber decrease the purity of the as-deposited metal nanoclusters [33]. The chemical composition reported for Pt deposited by FEBID varies from one study to another. Pt relative concentration in the range of 85% to 30% has been reported [40–41]. Since the chemical composition of the as-deposited nanostructures is closely related to their properties, such as electrical conductivity [17] or catalytic activity, the purity of the as-deposited nanoclusters is one of the main concerns in FEBID.

In order to improve the Pt purity in FEBID nanostructures, post-treatment such as annealing or using different precursors [33] has been developed. It has been shown that electron irradiation in SEM can improve the crystallinity and conductivity in the as-deposited Pt nanoclusters [42–43]. Room-temperature phase transformation is also obtained by using low-energy electron irradiation [44]. Another alternative to improve the crystallinity of as-deposited nanostructures is to use higher energy electron irradiation in TEM, with 200 kV electrons used to remove the amorphous carbon [32]. In this context, we used the electron beam in a TEM to reduce the amorphous carbon observed in the as-deposited cluster. The in-situ TEM irradiation has the advantage of site-specificity with simultaneous monitoring of the process.

**Figure 7:**
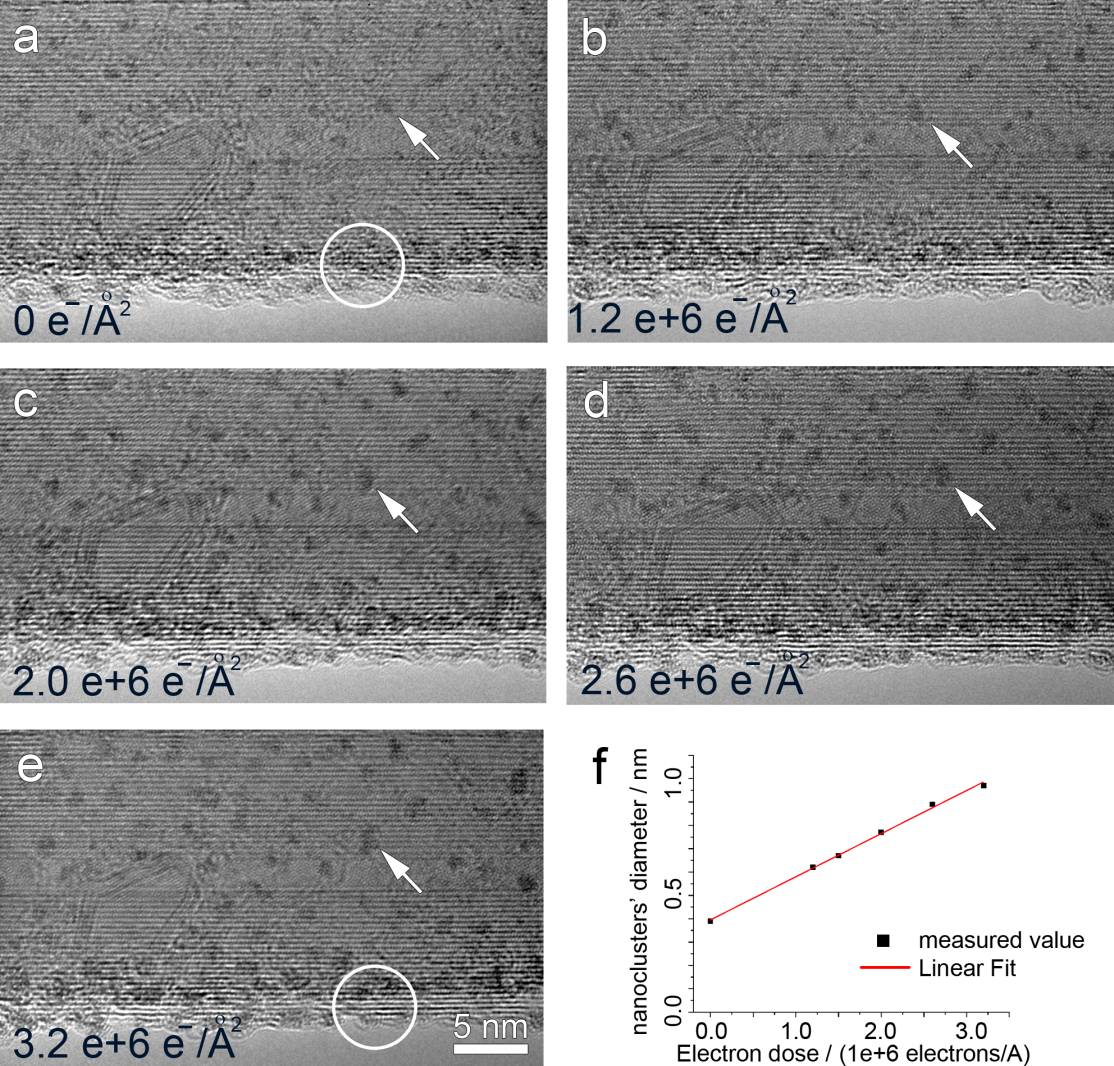
HRTEM images showing that amorphous carbon can be reduced by in-situ electron irradiation in a TEM under 80 kV. (a) shows the FEBID on CNT before electron irradiation. (b–e) show that the amorphous carbon is gradually cleaned by increasing the dose of electrons. Comparing the circled area indicated in (a) and (e), the cleaning of amorphous carbon is obvious. (a–e) also show the growing of nanoclusters by increasing electron dose, one example is indicated by an arrow through the image series. The growth of nanoclusters is quantified as shown in (f), with the diameter of nanoclusters increasing linearly by electron dose.

The electron irradiation in TEM was performed at an accelerating voltage of 80 kV, in order to minimize damage to the CNTs. Figure 7 presents the evolution of the FEBID nanostructures during the irradiation process. Figure 7a shows the as-deposited nanostructure before irradiation, where amorphous carbon is present on the CNT surface. Pt nanoclusters (dark contrast) are embedded within an amorphous carbon layer. After irradiation with a dose of 1.2 × 10^6^ electrons/Å^2^ (Figure 7b), 2.0 × 10^6^ electrons/Å^2^ (Figure 7c), 2.6 × 10^6^ electrons/Å^2^ (Figure 7d) and finally 3.2 × 10^6^ electrons/Å^2^ (Figure 7e), the amorphous layer is gradually reduced, as seen by comparing the same area from Figure 7a and Figure 7e as indicated by the circles, whereas the nanostructure of the CNT is well preserved. This effect has been reported in [45], which is attributed to the dissociation of intermediate amorphous carbon and defined as the first phase in electron irradiation. The second phase of graphitization due to the increasing electron–carbon interactions [43,45] can be observed from Figure 7e, where thin graphitic layers have emerged at the CNT surface, adding to the nondamaged CNT nanostructure.

Accompanying the reduction of amorphous carbon, the growth of Pt nanoclusters is noticed. As indicated by the arrows in Figure 7a to Figure 7e, a nanocluster is randomly picked to demonstrate the growth. It is likely that the growth of nanoclusters is due to the aggregation of Pt when the intermediate amorphous carbon is gradually removed. The growth of Pt nanoclusters under electron irradiation has been quantified as shown in Figure 7f. The average diameter of Pt nanoclusters are measured and plotted against electron dose, which shows a linear increase in the range of 0 to 3.2 × 10^6^ electrons/Å^2^.

A recent study has shown that FEBID of amorphous carbon on CNT followed by annealing of the structure, can result in a low-resistance electrical contact between CNT and metals, thanks to graphitic carbon layers crystallized from amorphous carbon [46]. Nevertheless, in the deposition of metal nanoclusters by FEBID, conventional annealing of the composite structure may lead to unwanted fast growth of nanoparticles, which can be difficult to monitor and control. Our post-growth experiment through electron irradiation has shown that the crystallization of amorphous carbon can be performed in a controllable manner, where the growth of nanoparticles is seen to increase along with the electron dose, and the graphitization of the carbon layer is present to reduce the resistance between the CNT and the deposited metal effectively.

In summary, focused-electron-beam deposition of Pt nanoclusters on CNTs in a DualBeam system has been demonstrated to be a viable approach to fabricate novel nanostructures. The deposition can be performed in a site-specific manner where the nanoclusters are strictly deposited in the area of interest whereas the rest of the CNT is free of modification and well preserved. Electron tomography is performed in order to reveal the 3D structure of the as-deposited CNTs. It has been found that the deposition takes place all over the CNT, resulting in Pt nanoclusters over the entire surface of the CNT. Some characteristics of the as-deposited nanostructures can be controlled by tuning the electron-beam parameters. Defocus in the electron beam leads to stripe patterning of the Pt nanoclusters across the CNTs. The ability to fine-tune the deposition of ultrasmall nanoclusters with a regular spacing on the nanoscale opens-up the possibility to engineering specific surface electronic states and, thus, catalytic activity.

The distribution of the as-deposited nanoclusters is closely related to the beam primary energy and dwell time during the deposition process. An electron beam of low primary energy has a significant influence on increasing the density of the as-deposited nanoclusters. Furthermore, the Pt purity of the as-deposited nanoclusters can be efficiently improved by reducing amorphous carbon using in-situ electron-beam irradiation in TEM at low kilovolt potentials (80 kV), where a simultaneous growth of a thin graphitic layer and Pt nanoclusters is achieved. The controllable crystallization of amorphous carboneous and Pt nanoclusters could be interesting for contact studies of functionalized CNTs.

## Experimental

CNTs supported on a TEM grid are used as the deposition target. The samples are prepared by using commercially available multiwalled carbon nanotubes (MWCNTs) produced by arc discharge or chemical vapor deposition (CVD). The CNTs powder is sonically dispersed in ethanol and then a drop of the solution is deposited onto a holey carbon film supported by a standard TEM copper grid.

FEBID (Figures 1, 3, 5) is performed by using an FEI Nova 200 Nanolab DualBeam SEM/FIB. During the deposition in the DualBeam system, the working distance is set to be 5.0 mm throughout the experiments. The DualBeam system is equipped with a standard gas injection system (GIS) with (CH_3_)_3_Pt(CpCH_3_) as organometallic precursor gas. The reservoir temperature was approximately 43 °C. The electron beam used for deposition can be accelerated between 3 kV and 30 kV with a beam current ranging from 44 pA to 4.3 nA. The electron dose for all experiments is maintained as 2.5 × 10^9^ electrons/μm^2^.

FEBID (Figures 4, 6, 7) is performed by using an FEI Helios DualBeam SEM/FIB. During the deposition in the DualBeam system, the working distance is set to be 4.0 mm throughout the experiments. The DualBeam system is equipped with a GIS with the same organometallic precursor gas of (CH_3_)_3_Pt(CpCH_3_). The electron beam used for deposition can be accelerated between 1 kV and 30 kV with varying beam current. The electron dose for Figure 4 was set to be at 8.5 × 10^8^ electrons/μm^2^, whereas the electron dose for Figure 6 and Figure 7 was maintained at 2.5 × 10^9^ electrons/μm^2^. The serpentine raster strategy is applied through all experiments, whereas the refresh time is 0 by default.

The as-deposited nanostructures are studied by using an FEI Tecnai G2 microscope operated at 200 kV. In order to investigate the morphology and distribution of the nanostructures in 3D, high-angle annular dark-field scanning transmission electron microscopy (HAADF-STEM) tilt series of the as-deposited CNTs are acquired. The tilt series have an angular range of ±70° with projections taken every 2°, and the tilt axis is set to be parallel to the long axis of the CNTs. Alignment of the tilt series is done by using the FEI Inspect3D software. The same software is used to reconstruct the aligned tilt series through the simultaneous iterative reconstruction technique (SIRT). The volume reconstructed by SIRT is segmented manually and visualized in the Amira software. High-resolution TEM (HRTEM) of the as-deposited nanostructures is performed on the same microscope at 200 kV. Post treatment using electron-beam irradiation is, however, performed using a FEI Titan 80–300 microscope fitted with aberration-correctors for the imaging lens and the probe forming lens, operated at 80 kV.

## Supporting Information

File 1 Reconstructed movie of CNT fully covered by Pt.

File 2 Detailed deposition parameters.

File 3 Reconstructed movie of CNT covered by stripe-patterned Pt.
