# Supplementary material for: Low-dose patterning of platinum nanoclusters on carbon nanotubes by focused-electron-beam-induced deposition as studied by TEM
Source: Beilstein J Nanotechnol. 2013 Feb 4;4:77–86. doi: 10.3762/bjnano.4.9 (PMC3566795; doi:10.3762/bjnano.4.9)
Supplement: File 2 — Detailed deposition parameters. [file Beilstein_J_Nanotechnol-04-77-s002.pdf]

Supporting Information

for

Low-dose patterning of Pt nanoclusters on carbon nanotubes by focused-electron-beam-induced deposition as studied by TEM

Xiaoxing Ke *[1], Carla Bittencourt [2], Sara Bals [1] and Gustaaf Van Tendeloo [1]

[1]EMAT, University of Antwerp, Groenenborgerlaan 171, 2020 Antwerp, Belgium and [2]Chemistry of Interaction Plasma Surface (ChiPS), University of Mons, Place du Parc 20, 7000 Mons, Belgium

Email: Xiaoxing Ke – Xiaoxing.Ke@ua.ac.be

* Corresponding author

Detailed deposition parameters

Detailed deposition parameters

Table S1: Detailed deposition parameters for Figure 4.

	a	b	c	d
Beam voltage	30 kV	30 kV	30 kV	30kV
Beam current	0.2 nA	0.2 nA	0.2 nA	0.2nA
Defocus value	0 μm	4 μm	8 μm	10 μm
pitch	2.5 nm	17.6 nm	35.0 nm	43.7nm
Loop time	168.1 ms	3.3 ms	843 μs	554μs
passes	2	103	412	638
blur	0 nm	17.5 nm	34.9 nm	43.7nm

Table S2: Detailed deposition parameters for Figure 6.

Beam current = 0.2 nA	Dwell time = 50 ns	Dwell time = 100 ns	Dwell time = 500 ns	Dwell time = 1 μs	Dwell time = 10 μs
Beam voltage = 1 kV, pitch = 19.68nm	t_{loop} = 134.6μs, Passes = 13800	t_{loop} = 267.2μs, Passes = 6908	N/A	t_{loop} =2.7 ms, Passes = 692	t_{loop} = 26.5ms, Passes = 69
Beam voltage = 3kV, pitch = 8.03nm	t_{loop} = 847μs, Passes = 2385	t_{loop} = 1.7ms, Passes = 1194	t_{loop} = 8.5ms, Passes = 239	t_{loop} = 16.9ms, Passes = 119	t_{loop} = 160ms, Passes = 12
Beam voltage = 5kV, pitch = 6.22nm	t_{loop} = 1.4ms, Passes = 1200	t_{loop} = 2.7 ms, Passes = 600	t_{loop} = 13.5ms, Passes = 120	t_{loop} = 26.9ms, Passes = 60	t_{loop} = 269ms, Passes = 6
Beam voltage = 10 kV, pitch = 4.4nm	t_{loop} = 3ms, Passes = 600	t_{loop} = 6.1ms, Passes = 300	t_{loop} = 30.4ms, Passes = 60	t_{loop} = 60.8ms, Passes = 30	t_{loop} = 607.6ms, Passes = 3
Beam voltage = 15 kV, pitch = 3.6nm	t_{loop} = 4.4ms, Passes = 450	t_{loop} = 7.9ms, Passes = 260	t_{loop} = 44.5ms, Passes = 45	t_{loop} = 79ms, Passes = 26	t_{loop} = 789.6 ms, Passes = 3
Beam voltage = 30 kV, pitch = 2.5nm	t_{loop} = 8.4ms, Passes = 238	t_{loop} = 16.8ms, Passes = 120	t_{loop} = 84ms, Passes = 24	t_{loop} = 168.1ms, Passes = 12	t_{loop} = 2s, Passes = 1
